# Shp-2 contributes to anti-RSV activity in human pulmonary alveolar epithelial cells by interfering with the IFN-α-induced Jak/Stat1 pathway

**DOI:** 10.1111/jcmm.12629

**Published:** 2015-06-27

**Authors:** Saisai Wang, Gang Zheng, Lifang Zhao, Feng Xu, Jing Qian

**Affiliations:** a Department of Medical Microbiology and Parasitology, Research Center of Infection and Immunity, Collaborative Innovation Center for Diagnosis and Treatment of Infectious Diseases, Zhejiang University School of MedicineHangzhou, Zhejiang, China; b Cancer Institute, Key Laboratory of Cancer Prevention and Intervention, China National Ministry of Education, Second Affiliated Hospital, Zhejiang University School of MedicineHangzhou, Zhejiang, China; c Department of Infectious Diseases, Second Affiliated Hospital Zhejiang University School of MedicineHangzhou, Zhejiang, China

**Keywords:** respiratory syncytial virus, Src homology phosphotyrosyl phosphatase 2, interferon, Jak/Stat

## Abstract

Src homology phosphotyrosyl phosphatase 2 (Shp-2) is a ubiquitously expressed protein that is involved in a variety of cellular processes, including antiviral interferon signalling pathways. In this study, we investigated the role of Shp-2 in the host cell interactions of human respiratory syncytial virus (RSV). We report significant changes in the expression of Shp-2 in human pulmonary alveolar epithelial cells (A549) upon RSV infection. We also report that blocking Shp-2 does not affect viral replication or virus-induced interferon-alpha (IFN-α) production. Interestingly, whereas A549 cells were activated by IFN-α, the blocking of Shp-2 resulted in increased viral replication that was associated with the reduced expression of the IFN-stimulated genes of 2′,5′-oligoadenylate synthetases and Mx1, and the concomitant inhibition of Stat1 tyrosine phosphorylation. Our findings suggest that Shp-2 contributes to the control of RSV replication and progeny production in pulmonary alveolar epithelial cells by interfering with IFN-α-induced Jak/Stat1 pathway activation rather than by affecting the production of IFN-α itself.

## Introduction

Human respiratory syncytial virus (RSV) is an enveloped negative-sense, single-stranded RNA virus of the *Paramyxoviridae* family. Respiratory syncytial virus infections are related to severe lower respiratory tract inflammation in infants and young children 1, particularly for those born preterm 2. To date, there is no specific chemotherapy or licensed vaccine available for RSV 3,4. The pathogenesis of RSV and the interacting between the virus and host immune responses appear to be complicated and are not fully understood.

Type I interferons (IFNs), primarily IFN-α/β, are produced by host cells as ‘early’ antiviral agents 5,6 and are recognized as a critical part of the host innate immune response to virus infection. In general, the binding of IFN-α/β to their receptors results in the cross-phosphorylation of Janus kinases (Jaks) at tyrosines, which provides docking sites for signal transducers and activators of transcription (Stats) leading to Stat phosphorylation. The phosphorylated Stats (pStats) then dissociate from the receptor, dimerize and translocate into the nucleus to regulate downstream gene expression 7. The IFN signalling cascade affects the expression of a large number of IFN-stimulated genes (ISGs), including ‘classical ISGs’, such as serine/threonine protein kinase, 2′,5′-oligoadenylate synthetases (2′,5′-OAS), Mx1 or the more recently identified apolipoprotein B mRNA editing enzyme, catalytic polypeptide-like 3G (APOBEC3G), ISG15 ubiquitin-like modifier (ISG15), adenosine deaminase, RNA-specific (ADAR), interferon induced transmembrane protein 1/2/3 (IFITM1/2/3) 8,9. With regards to RSV, respiratory epithelial cells are the cells first exposed to RSV infection and for host innate immune responses. The infection of RSV triggers toll-like receptor (TLR)3, TLR4, TLR7, TLR9 10–12 or retinoic acid-inducible gene I (RIG-I) 13 and stimulates cells to produce type I IFNs (IFN-α/β). Apparently, RSV has developed several ways to antagonize IFNs 14,15, and the viral nonstructural (NS) proteins NS1 and NS2 might be directly responsible for antagonizing IFN-α-associated signalling pathways 9,16,17. On the other hand, cellular factors are also involved in regulating IFN signalling pathways. For example, members of the suppressor of cytokine signalling (SOCS) family could be utilized by RSV and are involved in a feedback loop that inhibits cytokine responses and block the activation of Jak/Stat 18.

Src homology phosphotyrosyl phosphatase 2 (Shp-2), a member of the protein tyrosine phosphatase family, is a ubiquitously expressed phosphatase. This protein plays an important regulatory role in signalling equilibrium to control cellular responses and function. A variety of intracellular signal transduction pathways *i.e*., growth factors (including platelet-derived growth factor, epidermal growth factor and insulin-like growth factor 1), cytokines [including interleukin (IL)-3, granulocyte macrophage-colony stimulating factor, eosinophil peroxidase] and IFNs, are involved 19,20. Depending on the characteristics of a given extracellular stimulus, Shp-2 may negatively or positively interfere with Jak/Stat signalling cascades 21. For instance, Shp-2 negatively interferes with lipopolysaccharide-induced Toll/IL-1 receptor domain-containing adaptor inducing IFN-beta (TRIF) adaptor protein-dependent type I IFN production 22 although it positively regulates growth factor-dependent Jak/Stat5 activation in haematopoiesis 23. Despite the critical regulatory role of Shp-2 in intracellular IFN downstream signalling, whether it contributes to host IFN pathways to control RSV replication is not defined yet.

In this study, we dissected the expression pattern of Shp-2 in RSV-infected A549 human pulmonary alveolar epithelial cells and explored its role in controlling RSV replication and interfering with IFN-α production and function. We reveal that inhibition of Shp-2 leads to stronger RSV propagation in A549 cells only when cells are pre-activated with IFN-α. We also provided evidence that Shp-2 contributes to the anti-RSV activity of A549 cells by interfering with the IFN-α-induced Jak/Stat1 pathway and ISG expression.

## Materials and methods

### Cells and chemicals

Human epithelial cell type 2 (HEp-2) cells from the American Tissue Culture Collection (ATCC, CCL-23^–^) were grown in RPMI1640 medium supplemented with 10% heat-inactivated foetal calf serum (FBS), 2 mM glutamine and 100 units/ml penicillin/streptomycin. Human pulmonary alveolar epithelial cells (A549) obtained from ATCC (CRM-CCL-185^–^) were cultured in DMEM with the supplements listed above. Recombinant human IFN-α (rh IFN-α) and phenyl hydrazono pyrazolone sulphonate 1 (PHPS1) were purchased from Peprotech (300-02AA; Rocky Hill, NJ, USA) and Sigma-Aldrich (P0039; St. Louis, MO, USA), respectively. PHPS1 was dissolved in DMSO. All other cell culture reagents were from Invitrogen (Shanghai, China).

### Viruses

The long strain of RSV was obtained from ATCC (VR-26^–^) and propagated in HEp-2 cells. The virus was grown in confluent HEp-2 cells in 75 cm^2^ flasks at a multiplicity of infection (moi) of 0.05 in tissue culture medium consisting of RPMI1640 supplemented with 2% heat-inactivated FBS. When the cytopathic effects reached 80–90%, the entire culture (including cells and supernatant) was frozen at −80°C followed by three runs of thawing and freezing. The cells and supernatant were collected and centrifuged at 2000 × g for 15 min. at 4°C to clear cell debris. The resulting supernatant containing viruses were collected for ultracentrifugation at 50,000 × g for 1 hr at 4°C. The remaining pellet was resuspended in 1 ml serum-free RPMI1640, divided into aliquots and stored at −80°C. The titre was determined by methylcellulose plaque assay on HEp-2 cells.

### Infection of epithelial cells and sampling

In this study, human pulmonary alveolar epithelial cells (A549) were used as an epithelial cell model for RSV infection. Briefly, cells were absorbed at 37°C with RSV at a moi of 1 and gently rocked every 15 min. After 1 hr, the virus inoculum was removed, and the cells were washed and cultured with fresh medium. At the indicated times post infection, cells were harvested for the extraction of RNA or protein, the cell culture supernatants were collected for ELISA, and the entire culture was harvested for plaque assay. Uninfected cells served as negative controls. Where indicated, A549 cells were also incubated with ultraviolet (UV)-inactivated RSV (non-replicating virus) as an additional control.

For IFN-α treatment, the cell culture media was replaced by medium with or without 100 U/ml IFN-α at 6 hrs before RSV infection. For Shp-2 inhibition, PHPS1 or DMSO (solvent control) was added to the cell cultures to the indicated final concentrations at 30 min. before RSV infection.

### IFN-α ELISA

The IFN-α concentration was assessed with the IFN-α ELISA kit (Dakewei Biotech Co., Ltd., Shanghai, China) according to the manufacturer’s instructions.

### Plaque assay

Virus titre was determined by the plaque assay. Briefly, 200 μl of 10-fold serially diluted virus stocks or cell-free supernatants were incubated with HEp-2 monolayers in duplicate for 1 hr. After incubation, cells were overlaid with 1% (wt/vol) methylcellulose containing 50% (vol/vol) 2× DMEM and 2% (vol/vol) FBS and cultured for another 5 days before the overlay medium was removed, and cells were fixed and stained with 2% (wt/vol) crystal violet in 20% (vol/vol) ethanol. Wells containing of 30–100 plaques were counted and the viral titre was calculated using the following formula: virus titre (pfu/ml) = Plaques × dilution × 5.

### Quantitative real-time PCR (RT-qPCR)

Total RNA was extracted with the RNA simple Total RNA Kit (Tiangen, Beijing, China). First-strand cDNA was synthesized from 1 μg total RNA (DNase-treated) using the iScript cDNA synthesis kit (Bio-Rad, Richmond, CA, USA). Reverse transcriptase PCR was performed with a 2720 Thermal Cycler (Applied Biosystems, Paisley, UK). For each sample, a no reverse-transcriptase reaction was performed to serve as a control to exclude the existence of endogenous genomic DNA. qPCRs were then performed with 1 μl cDNA using iTaq Universal SYBR Green Supermix (Bio-Rad) with the CFX96 Touch Real-Time PCR Detection System (Bio-Rad). SYBR green PCR was run at 95 for 3 min. and 35 cycles of 95 for 30 sec., 55 for 30 sec., and 72 for 30 sec. followed by melting curve analysis. Primers sequences for RSV-F (RSV F protein), hIFN-α, 2′,5′-OAS1, Mx1, Shp-2, and β-actin are listed in Table 1. qPCR results were analysed with Bio-Rad CFX Manager. A relative quantification strategy was used to analyse qPCR data. The calculated threshold cycle was normalized to the value of the internal control β-actin amplified from the same samples, and the fold-change compared with control was calculated based on the delta-delta Ct method.

**Table 1:** Primer sequences for target genes

Gene	Forward primer	Reverse primer
β-actin	GTATCCTGACCCTGAAGTACC	TGAAGGTCTCAAACATGATCT
RSV-F	TTGGATCTGCAATCGCCA	CTTTTGATCTTGTTCACTTCTCCTTCT
IFN-α	GCCTCGCCCTTTGCTTTACT	CTGTGGGTCTCAGGGAGATCA
2′,5′-OAS1	TGTCCAAGGTGGTAAAGGGTG	CCGGCGATTTAACTGATCCTG
Mx1	AGCGGGATCGTGACCAGAT	TGACCTTGCCTCTCCACTTATC
Shp-2	GACATACCACTTTCGGACCT	TGATGCTCTCCTGCTTATCG

### Western blotting

Cells were lysed in 1× RIPA buffer (CST, Danvers, MA, USA) containing 1 mM phenyl methyl sulphonyl fluoride. Protein concentrations were determined by the Bradford protein assay. A total of 30 μg of total proteins was separated in a 10% SDS-PAGE gel and transferred to PVDF membranes (Millipore, Billerica, MA, USA). Membranes were blocked with blocking buffer (Beyotime, Jiangsu, China) for 1 hr and then incubated overnight at 4°C with antibodies directed against phospho-Stat1 (Tyr701; 9167; CST, Stat1; 9172; CST), Shp-2 (sc-7384; Santa Cruz Biotechnology, Santa Cruz, CA, USA) and β-actin (4967; CST), respectively. After washing, the membranes were probed with a horse radish peroxidase-conjugated goat anti-rabbit secondary antibody (Lianke, Hangzhou, China) followed detection of signals with the FluorChem E System (Protein Simple, Santa Clara, CA, USA). The relative density of each band was measured with Image J.

### Statistical analysis

All assays were performed in triplicate, and experiments were repeated at least three times. Stata 9.1 software (Stata Corp., College Station, TX, USA) was used to process the data. Data are presented as the means ± SEM. Significant differences between two groups were determined by Student’s *t*-test with significance set at *P* < 0.05.

## Results

### RSV infection alters Shp-2 expression in A549 human pulmonary alveolar epithelial cells

**Figure 1:**
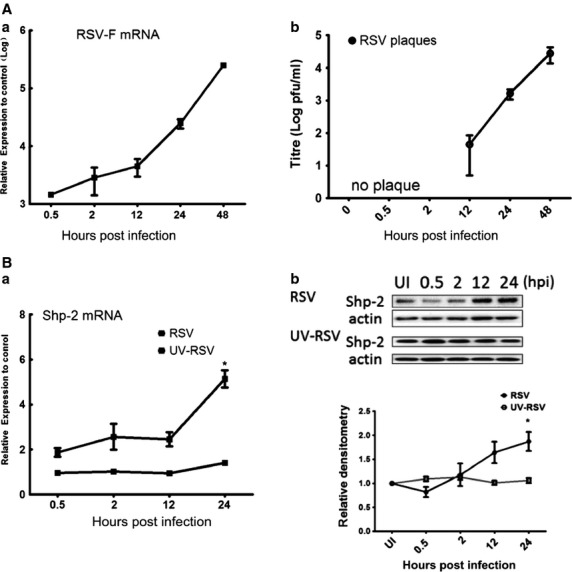
Infection with RSV alters Shp-2 expression in A549 human pulmonary alveolar epithelial cells. A549 cells were infected with RSV or UV-RSV (non-replicating virus) at moi = 1. At the indicated time-points post infection, (A) total RNA was extracted for RT-qPCR to evaluate RSV-F mRNA expression or cell cultures were collected for plaque assays to determine the virus titre. (B) Total RNA was extracted for RT-qPCR to evaluate Shp-2 mRNA expression, or protein was extracted for western blotting. Membranes were incubated with primary antibodies (diluted 1:1000 for β-actin and 1:500 for Shp-2). A representative immunoblot image is shown. Densitometry was performed on three separate experiments, and the graphs show the fold increase. An uninfected sample (UI) was considered as a control. *P < 0.05 *versus* control.

First, we examined the patterns of virus replication and Shp-2 expression in RSV-infected A549 human pulmonary alveolar epithelial cells. After infection with RSV or UV-RSV at a moi of 1 for 1 hr, cells were allowed to grow for the indicated period before they were harvested for Shp-2 western blotting and RT-qPCR. In parallel, similar cells were harvested for the detection of the viral titter and viral RSV-F mRNA expression. As indicated in Figure 1A, the expression of RSV-F mRNA could be detected as early as half 1 hr post infection (hpi), and the level increased by a log at 12 hpi and reached its maximum at 48 hpi. Viral plaque formation could only be detected after 12 hpi, but the expression pattern was similar to that of RSV-F mRNA expression. RT-qPCR results revealed that there was endogenous Shp-2 expression in uninfected A549 cells. The expression pattern of Shp-2 after RSV infection was similar to that of the RSV-F mRNA and viral plaque formation. At 24 hrs after RSV infection, the expression of Shp-2 was significantly increased. The increased expression of Shp-2 appeared to require virus replication because UV-RSV had no effect on Shp-2 expression (Fig. 1B). Western blotting of Shp-2 and density analyses revealed that there was endogenous expression of Shp-2 in A549 cells, which significantly increased at 24 hpi of RSV compared with uninfected cells. The infection of UV-RSV failed to increase its expression (Fig. 1B). Based on the expression characteristics of RSV-F mRNA and Shp-2 mRNA/protein, 12 and 24 hpi were selected for subsequent experiments.

### Shp-2 does not interfere with the virus replication or IFN-α production of RSV-infected A549 human pulmonary alveolar epithelial cells

**Figure 2:**
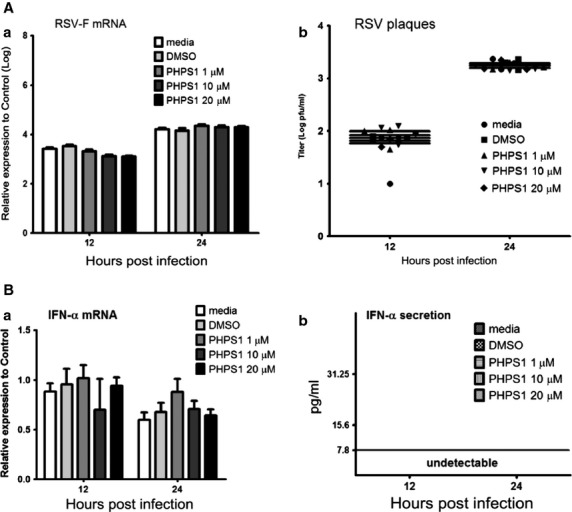
Shp-2 has no effect on IFN-α production and virus replication in RSV-infected A549 human pulmonary alveolar epithelial cells. A549 cells were pre-treated with DMSO or PHPS1 at 1, 10, 20 μM for 30 min. and then infected with RSV at moi = 1. At 12 and 24 hpi, (A) total RNA was extracted for RT-qPCR to evaluate RSV-F expression, and cells were collected for plaque assays to determine the virus titre. (B) Total RNA was extracted for RT-qPCR to evaluate the IFN-α mRNA expression, and cell supernatants were collected for ELISA to determine the IFN-α production.

Next, we explored whether Shp-2 plays a role in the anti-RSV activity and the IFN production of human pulmonary alveolar epithelial cells. The chemical antagonist PHPS1, which is a widely used cell-permeable inhibitor that binds to active center of Shp-2 24 and thus effectively blocks its function. To our surprise, pre-treatment of A549 cells with PHPS1 prior to RSV infection did not affect RSV replication as indicated by RSV-F mRNA and RSV titre (Fig. 2A) and the mRNA level of IFN-α (Fig. 2B). The secretion of IFN-α was under the detection level for the ELISA kit. Concentrations of PHPS1 ranging from 1 to 20 μM were used, but the results remained the same.

### Shp-2 interferes with the IFN-α-induced anti-RSV activity of A549 human pulmonary alveolar epithelial cells

**Figure 3:**
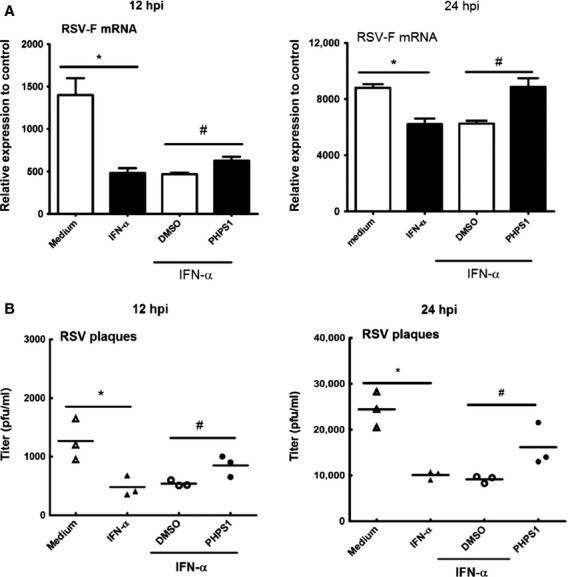
Shp-2 involves in the IFN-α triggered anti-RSV activity of A549 human pulmonary alveolar epithelial cells. 100 U/ml rhIFN-α or medium was applied to the A549 cell cultures 6 hrs prior to RSV infection. Treatment with 10 μM PHPS1 or DMSO and the infection of RSV were performed as described in Figure 2. At 12 hpi and 24 hpi, (A) total RNA was extracted and RT-qPCR was performed to evaluate RSV-F expression, (B) total cell culture (cells and cell culture supernatants) were collected for plaque assay to determine the RSV titres. The uninfected sample (UI) was considered as control. *P < 0.05 compared to medium control group. ^#^*P* < 0.05 compared to DMSO group.

Although RSV-infected cells do not appear to produce sufficient IFN-α, cells may be partially responsive to IFN-α treatment 25,26. We next looked to determine whether Shp-2 is involved in the IFN-α-induced anti-RSV response. In this regard, A549 cell cultures were treated with 100 U/ml rhIFN-α for 6 hrs to fully activate the IFN downstream pathway before RSV infection with or without PHPS1 treatment. Cells were collected at 12 and 24 hpi, and virus replication and IFN production were measured. Figure 3 shows that IFN-α treatment reduces the level of RSV replication in A549 cells. Interestingly, the treatment with PHPS1 attenuated the anti-RSV effects of IFN-α in A549 cells to a significant degree. These results strongly suggest that Shp-2 affects the downstream IFN-α signalling cascade rather than the production of IFN-α in RSV-infected human pulmonary alveolar epithelial cells.

### Shp-2 interferes with ISG expression

**Figure 4:**
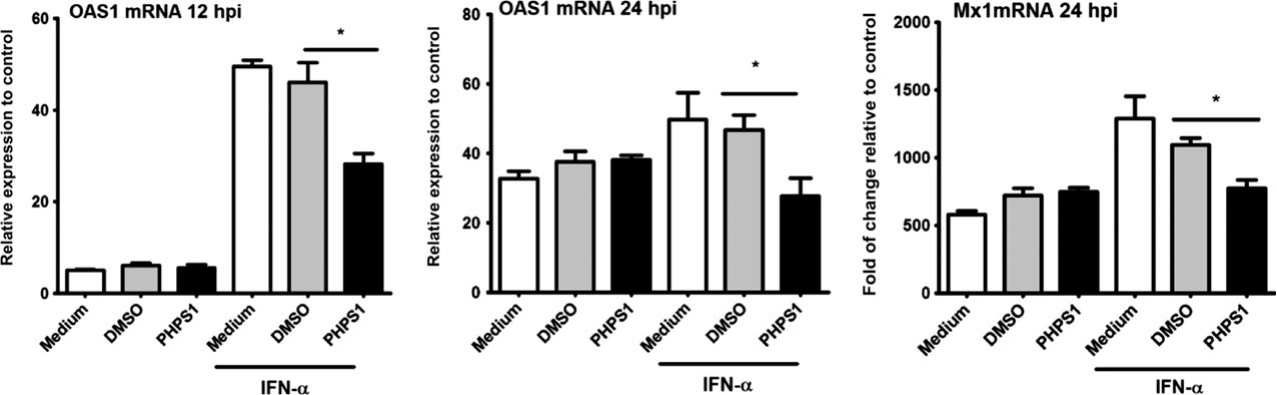
Inhibition of Shp-2 led to a decrease in IFN-α-induced antiviral gene expression in RSV-infected A549 human pulmonary alveolar epithelial cells. Treatment with rhIFN-α and PHPS1 and infection with RSV were performed as described in Figure 3. At 12 and 24 hpi, cells were collected for RNA extraction, and RT-qPCR was performed to evaluate the expression of 2′,5′-OAS1 and Mx1. An uninfected sample (UI) was considered as control. *P < 0.05 compared with DMSO.

To further demonstrate that Shp-2 interferes with the downstream IFN-α pathway, we examined the expression of 2′,5′-oligoadenylate synthetase1 (2′,5′-OAS1) and Mx1, the two ISGs significant for their antivirus activities. As shown in Figure 4, RSV infection could directly induce the expression of 2′,5′-OAS1 and Mx1, while there was no significant difference the between DMSO and PHPS1 groups. However, after IFN-α treatment, the expression of 2′,5′-OAS1 and Mx1 was significantly down-regulated in A549 cells treated with PHPS1. Thus, ISG expression is at least partially dependent on functional Shp-2.

### Shp-2 interferes with IFN-α-induced Stat1 phosphorylation

**Figure 5:**
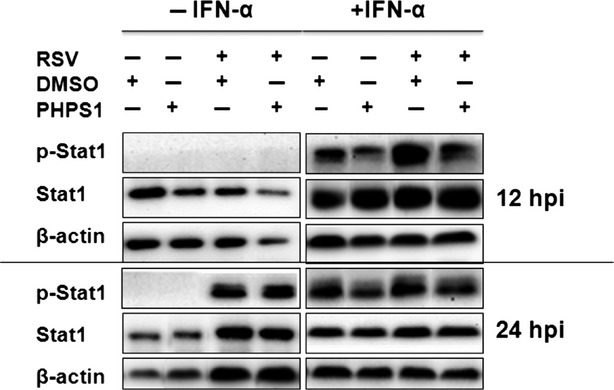
Inhibition of Shp-2 decreases IFN-α-induced STAT1 tyrosine phosphorylation. Treatment with rhIFN-α and PHPS1 and RSV infection were performed as described in Figure 3. At 12 and 24 hrs post infection, cells were collected for protein extraction and western blotting. Membranes were probed with primary antibodies directed against p-Stat1, Stat1 and β-actin.

Because that functions of IFNs are regulated by the Jak signal transducer and Stat signalling cascade, we explored whether Shp-2 was involved in interactions with the Jak-Stat pathway by examining the tyrosine phosphorylation of Stat1, a key molecule that is indispensable for pathway activity. As detailed in Figure 5A, IFN directly induced the phosphorylation of Stat1 that was inhibited by PHPS 1. Respiratory syncytial virus infection could induce the phosphorylation of Stat1 at 24 hpi, nevertheless the presence of PHPS1 did not change Stat1 phosphorylation. With IFN-α treatment, the presence of PHPS1 attenuated the tyrosine phosphorylation of Stat1 at 12 and 24 hpi in RSV-infected A549 cells. Thus, in this context, Shp-2 regulates phosphorylated Stat1 in IFN pathway.

## Discussion

In this study, we found that there was endogenous and RSV-induced expression of Shp-2 in a human pulmonary alveolar epithelial cell line (A549). Shp-2 inhibition by the PHPS1 did not lead to changes in the production of RSV progeny and IFN-α expression. Considering that RSV is a poor inducer of IFN-α/β in HEp 27, we suggested that the effect of Shp-2 on IFN production, if any, is not significant for RSV-host cell interaction.

We observed the interesting phenomenon that treatment with PHPS1 leads to an increase in RSV progeny production when cells are pretreated with IFN-α. Interferon-α can activate the Jak/Stat1 signalling pathway and induce the expression of antiviral ISGs 28, thus,Stat1 activation plays an important role in establishing a protective immune response to RSV infection 28. Our data also showed that inhibition of Shp-2 was accompanied by inhibition of IFN-α-induced Stat1 tyrosine phosphorylation and the decreased expression of the antiviral genes 2′,5′-OAS1 and Mx1. These findings suggest that Shp-2 plays a positive role in the regulation of the IFN-α-triggered anti-RSV pathway in human pulmonary alveolar epithelial A549 cells.

**Figure 6:**
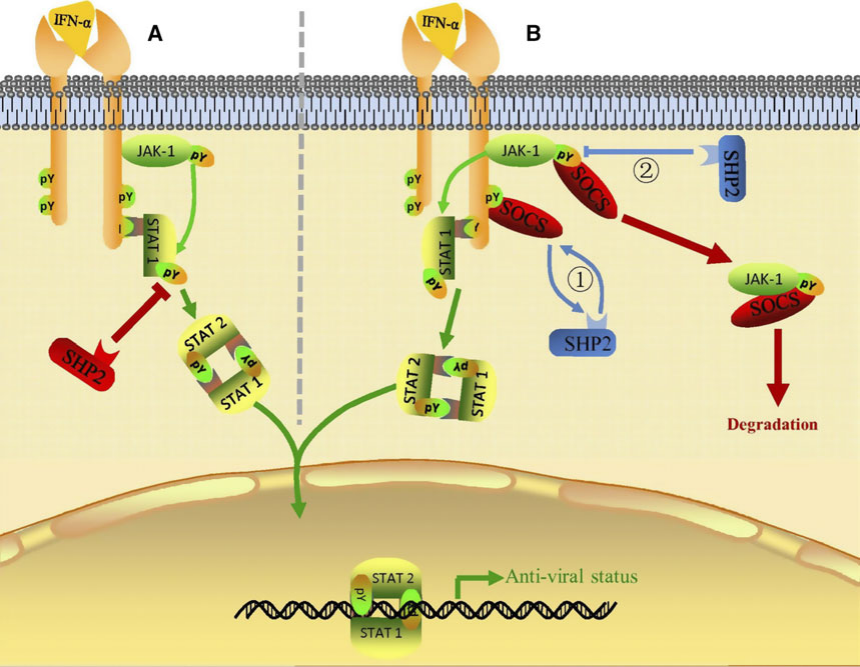
Novel mechanisms Shp-2 used to regulate the IFN-α-induced Jak/Stat1 signalling pathway. The classical IFN-α-induced Jak/Stat1 signalling pathway is shown in green: IFNα/β binds to IFNAR and forms an active complex. The binding of the intracellular domains of IFNAR with Jak1 results in the dimerization and rearrangement of receptor subunits, leading to the activation of associated Jaks by autophosphorylation and the further activation of Stat1 protein. Following phosphorylation, activated Stat1 forms heterodimers and then translocates into the nucleus where they initiate the transcription of IFN-stimulated genes. (A) Shp-2 is recognized as a negative player in the IFN-α-induced-Jak-Stat1 signalling pathway through its direct dephosphorylation of p-stat1. (B) Shp-2 might indirectly positively regulates the IFN-α-induced Jak-Stat1 pathway by interfering with SOCS by (1) Shp-2 competing with SOCS for the same binding site in IFNAR, possibly because of the same SH2 domain possessed by Shp-2 and SOCS, preventing the binding of SOCS proteins to IFNAR and the consequential down regulation of Jak/Stat activity and (2) Shp-2 dephosphorylating active Jak, which leads to the loss of a target site in Jak for SOCS proteins for degradation through the ubiquitin–proteasome pathway.

It is a widely accepted notion that Shp-2 is a negative regulator of the Jak-Stat1 signalling pathway 29 *via* its direct interaction with and de-phosphorylation of Stat1 at the Tyr701 and Ser727 residues 30 (illustrated in Fig. 6A). Indeed, it has been shown that Shp-2 is involved in the inhibition of IFN-α- and IFN-γ-induced Jak-Stat signalling pathway activation in the context of Japanese encephalitis virus or human cytomegalovirus infection 31,32. However, our results suggest an opposite functional role of Shp-2 which serves as a positive regulator of Jak-Stat1 signalling pathway and contributes to the anti-RSV activity of human epithelial host cells.

Shp-2 plays a positive regulatory role in several signalling pathways by regulating SOCS recruitment. SOCS proteins are considered negative regulators of cytokine receptor signalling 33,34 with its key structural features including an SH2 domain and a SOCS box 35,36. SH2 domains from SCOS3 and Shp-2 have similar binding specificities 37. In the IL-6 signalling pathway, Shp-2 competes with SOCS3 for binding to gp130 at the pY759 site and prevents downregulation of the receptor. Consequently, this mechanism leads to Stat3 activation 38,39. Up-regulation of SOCS1, SOCS3 and SOCS6 expression was previously reported in RSV-infected epithelial cells 18; therefore, we hypothesize that Shp-2 might compete with SOCS for the binding sites on tyrosine-phosphorylated IFN-α receptors, thus compensating for the inhibition effects of Stat1 signalling because of SOCS up-regulation (illustrated in Fig. 6B, pathway1).

It has been defined that SOCS inhibits Jak kinase activity by binding to the activation loop of the kinase and degrading it through the ubiquitin/proteasome pathway 33,34. Ali *et al*. demonstrated that in response to prolactin receptor activation, Shp-2 dephosphorylates Jak2 Y1007 to prevent the formation of a complex between Jak2 and SOCS-1, thus inhibiting the degradation of Jak by SOCS1 and maintaining the activity and stability of Jak2 40. We therefore hypothesize that a similar mechanism might underlie the activation of Jaks and the Jak/Stat1 pathway by Shp-2 (illustrated in Fig. 6B, pathway2).

In summary, we have described a novel role for Shp-2 as a positive regulator of the IFN-α downstream Jak/Stat1 signalling pathway, which thus contributes to the anti-RSV activity of epithelial cells upon IFN-α activation. This new bioactivity of Shp-2 may broaden our understanding of the interaction between RSV and host cell proteins and may aid in the design of effective antiviral therapeutic strategies. Combination of protein phosphatases with IFN-α or the early application of IFN-α might have the therapeutic potential to eliminate RSV infection. However, more studies are needed to explore the cellular targets of Shp-2 and the mechanisms that regulate the Jak/Stat pathway in primary cell culture of the air–liquid interface in the future experiments.

## References

[b1] Hall CB (2010). Respiratory syncytial virus in young children. Lancet.

[b2] Murray J, Bottle A, Sharland M (2014). Risk factors for hospital admission with RSV bronchiolitis in England: a population-based birth cohort study. PLoS ONE.

[b3] Guichelaar T, Hoeboer J, Widjojoatmodjo MN (2014). Impaired immune response to vaccination against infection with human respiratory syncytial virus at advanced age. J Virol.

[b4] Graham BS (2011). Biological challenges and technological opportunities for respiratory syncytial virus vaccine development. Immunol Rev.

[b5] Katze MG, He Y, Gale M (2002). Viruses and interferon: a fight for supremacy. Nat Rev Immunol.

[b6] Decker T, Stockinger S, Karaghiosoff M (2002). IFNs and STATs in innate immunity to microorganisms. J Clin Invest.

[b7] Kisseleva T, Bhattacharya S, Braunstein J (2002). Signaling through the JAK/STAT pathway, recent advances and future challenges. Gene.

[b8] de Veer MJ, Holko M, Frevel M (2001). Functional classification of interferon-stimulated genes identified using microarrays. J Leukoc Biol.

[b9] Schoggins JW, Wilson SJ, Panis M (2011). A diverse range of gene products are effectors of the type I interferon antiviral response. Nature.

[b10] Haynes LM, Moore DD, Kurt-Jones EA (2001). Involvement of toll-like receptor 4 in innate immunity to respiratory syncytial virus. J Virol.

[b11] Schlender J, Hornung V, Finke S (2005). Inhibition of toll-like receptor 7- and 9-mediated alpha/beta interferon production in human plasmacytoid dendritic cells by respiratory syncytial virus and measles virus. J Virol.

[b12] Rudd BD, Burstein E, Duckett CS (2005). Differential role for TLR3 in respiratory syncytial virus- induced chemokine expression. J Virol.

[b13] Liu P, Jamaluddin M, Li K (2007). Retinoic acid-inducible gene I mediates early antiviral response and Toll-like receptor 3 expression in respiratory syncytial virus-infected airway epithelial cells. J Virol.

[b14] Atreya PL, Kulkarni S (1999). Respiratory syncytial virus strain A2 is resistant to the antiviral effects of type I interferons and human MxA. Virology.

[b15] Ramaswamy M, Shi L, Monick MM (2004). Specific inhibition of type I interferon signal transduction by respiratory syncytial virus. Am J Respir Cell Mol Biol.

[b16] Schlender J, Bossert B, Buchholz U (2000). Bovine respiratory syncytial virus nonstructural proteins NS1 and NS2 cooperatively antagonize alpha/beta interferon-induced antiviral response. J Virol.

[b17] Hastie ML, Headlam MJ, Patel NB (2012). The human respiratory syncytial virus nonstructural protein 1 regulates type I and type II interferon pathways. Mol Cell Proteomics.

[b18] Hashimoto K, Ishibashi K, Ishioka K (2009). RSV replication is attenuated by counteracting expression of the suppressor of cytokine signaling (SOCS) molecules. Virology.

[b19] Huyer G, Alexander DR (1999). Immune signalling: SHP-2 docks at multiple ports. Curr Biol.

[b20] Qu CK (2000). The SHP-2 tyrosine phosphatase: signaling mechanisms and biological functions. Cell Res.

[b21] Xu D, Qu CK (2008). Protein tyrosine phosphatases in the JAK/STAT pathway. Front Biosci.

[b22] An H, Zhao W, Hou J (2006). SHP-2 phosphatase negatively regulates the TRIF adaptor protein-dependent type I interferon and proinflammatory cytokine production. Immunity.

[b23] Li L, Modi H, McDonald T (2011). A critical role for SHP2 in STAT5 activation and growth factor-mediated proliferation, survival, and differentiation of human CD34^+^ cells. Blood.

[b24] Hellmuth K, Grosskopf S, Lum CT (2008). Specific inhibitors of the protein tyrosine phosphatase Shp2 identified by high-throughput docking. Proc Natl Acad Sci USA.

[b25] Guerrero-Plata A, Baron S, Poast JS (2005). Activity and regulation of alpha interferon in respiratory syncytial virus and human metapneumovirus experimental infections. J Virol.

[b26] Chipps BE, Sullivan WF, Portnoy JM (1993). Alpha-2A-interferon for treatment of bronchiolitis caused by respiratory syncytial virus. Pediatr Infect Dis J.

[b27] Spann KM, Tran KC, Chi B (2004). Suppression of the induction of alpha, beta, and lambda interferons by the NS1 and NS2 proteins of human respiratory syncytial virus in human epithelial cells and macrophages. J Virol.

[b28] Durbin JE, Johnson TR, Durbin RK (2002). The role of IFN in respiratory syncytial virus pathogenesis. J Iimmunol.

[b29] You M, Yu DH, Feng GS (1999). Shp-2 tyrosine phosphatase functions as a negative regulator of the interferon-stimulated Jak/STAT pathway. Mol Cell Biol.

[b30] Wu TR, Hong YK, Wang XD (2002). SHP-2 is a dual-specificity phosphatase involved in Stat1 dephosphorylation at both tyrosine and serine residues in nuclei. J Biol Chem.

[b31] Lin RJ, Chang BL, Yu HP (2006). Blocking of interferon-induced Jak-Stat signaling by Japanese encephalitis virus NS5 through a protein tyrosine phosphatase-mediated mechanism. J Virol.

[b32] Baron M, Davignon JL (2008). Inhibition of IFN-gamma-induced STAT1 tyrosine phosphorylation by human CMV is mediated by SHP2. J Immunol.

[b33] Iwamoto T, Senga T, Naito Y (2000). The JAK-inhibitor, JAB/SOCS-1 selectively inhibits cytokine-induced, but not v-Src induced JAK-STAT activation. Oncogene.

[b34] Zhang JG, Farley A, Nicholson SE (1999). The conserved SOCS box motif in suppressors of cytokine signaling binds to elongins B and C and may couple bound proteins to proteasomal degradation. Proc Natl Acad Sci USA.

[b35] O’Shea JJ, Gadina M, Schreiber RD (2002). Cytokine signaling in 2002: new surprises in the Jak/Stat pathway. Cell.

[b36] Yoshimura A, Naka T, Kubo M (2007). SOCS proteins, cytokine signalling and immune regulation. Nat Rev Immunol.

[b37] De Souza D, Fabri LJ, Nash A (2002). SH2 domains from suppressor of cytokine signaling-3 and protein tyrosine phosphatase SHP-2 have similar binding specificities. Biochem.

[b38] Schmitz J, Weissenbach M, Haan S (2000). SOCS3 exerts its inhibitory function on interleukin-6 signal transduction through the SHP2 recruitment site of gp130. J Biol Chem.

[b39] Lehmann U, Schmitz J, Weissenbach M (2003). SHP2 and SOCS3 contribute to Tyr-759-dependent attenuation of interleukin-6 signaling through gp130. J Biol Chem.

[b40] Ali S, Nouhi Z, Chughtai N (2003). SHP-2 regulates SOCS-1-mediated Janus kinase-2 ubiquitination/degradation downstream of the prolactin receptor. J Biol Chem.

